# Etodolac improves collagen induced rheumatoid arthritis in rats by inhibiting synovial inflammation, fibrosis and hyperplasia

**DOI:** 10.1186/s43556-021-00052-1

**Published:** 2021-10-25

**Authors:** Qin Feng, Wenkai Xia, Shenglan Wang, Guoxin Dai, Weimei Jiao, Na Guo, Honghua Li, Guimin Zhang

**Affiliations:** 1 Lunan Pharmaceutical Group Co., Ltd., Linyi, China; 2 Center for New Drug Safety Evaluation of Lunan Pharmaceutical, Lunan Pharmaceutical Group Co. Ltd., Linyi, China; 3 National Engineering and Technology Research Center of Chirality Pharmaceutical, Lunan Pharmaceutical Group Co. Ltd., Linyi, China

**Keywords:** Rheumatoid arthritis, Synovial hyperplasia, Proteomics, Etodolac

## Abstract

Synovial hyperplasia is the main cause of chronic rheumatoid arthritis (RA), but the mechanism of synovial hyperplasia is still unclear. Etodolac (ETD) is a selective COX-2 inhibitor for relieving pain and stiffness in RA, but the disease modifying effect is still lack of evidence. Proteomics method was used to study the differential proteome of synovial tissue in collagen induced arthritis (CIA) in rats. With the help of STRING analysis, the upregulated proteins enriched in the cluster of complement and coagulation cascades and platelet degranulation were highlighted, these proteins with fibrogenic factors Lum, CIV, CXI and Tgfbi participated in the synovial inflammation, fibrosis and hyperplasia in CIA. Based on KOG function class analysis, the proteins involved in the events of the central dogma was explored. They might be hyperplasia related proteins for most of them are related to the proliferation of cancer. ETD significantly attenuated synovial inflammation, fibrosis and hyperplasia in CIA rats by downregulating these proteins. Several proteins have not been observed in RA so far, such as Tmsb4x, Pura, Nfic, Ruvbl1, Snrpd3, U2af2, Srrm2, Srsf7, Elavl1, Hnrnph1, Wars, Yars, Bzw2, Mcts1, Eif4b, Ctsh, Lamp1, Dpp7, Ptges3, Cdc37 and Septin9, they might be potentials targets for RA. Blood biochemistry tests showed the safety of 7 months use of ETD on rats. In conclusion, present study displayed a comprehensive mechanism of synovial hyperplasia in CIA rats, on this basis, the clinical value of ETD in the treatment of RA was well confirmed.

**Supplementary Information:**

The online version contains supplementary material available at 10.1186/s43556-021-00052-1.

## Instruction

RA is a chronic systemic autoimmune disease. It mainly invades joints all over the body, presenting multiple and symmetrical chronic proliferative synovitis, which leads to destruction of articular cartilage and joint capsule, and finally leads to ankylosis of joints [1]. In addition, it seriously affects the health of patients, causing persistent pain and leading to severe disability. The etiology of RA is still unclear. It is generally believed that genetic factors and environmental factors play important roles in the etiology of rheumatoid arthritis.

The key therapeutic goal of RA is remission, no active arthritis, no erosion or deterioration of function. Other important goals are to reduce disease activity and pain, maintain function, and retain work and recreational activities. Currently, glucocorticoids and non-steroidal anti-inflammatory drugs (NSAIDs) were used for the treatment of RA, mainly for the control of pain and inflammation, disease modifying anti rheumatic drugs, as the first-line drugs for the new diagnosis of RA, and biotherapy, which were used to target and inhibit specific molecules of immune and inflammatory response [2].

NSAIDs are used widely to control symptoms of RA for lessen pain and stiffness. It is well known that the quality of life in arthritis patients receiving NSAIDs has been improved significantly, but they have lost their historical role as first-line treatment because of concerns about their limited effectiveness, inability to modify the long-term course of disease, and gastrointestinal and cardiac toxic effects [3]. But the risks are different from the types of NSAIDs. And there are studies showing that the effects of NSAIDs are more than control symptoms but also have the effects of alleviating disease and delaying the progress of RA [4]. All above, the use of NSAIDs is still certainly needed, and it is very important to choose safe and effective NSAIDs for long-term use. The ideal NSAIDs should inhibit prostaglandin (PG) synthesis in the inflammatory site, not in the gastric mucosa. Experimental and clinical data support that ETD meets this standard. Long-term daily administration of ETD effective anti-inflammatory dose (rat 3 mg· kg^− 1^; 600 mg in humans) had no effect on PGF2 and prostacyclin in gastric mucosa. The selectivity of ETD to COX-2 was 10 times, and the retention of COX-1 activity in gastric mucosa led to a significant increase in gastric tolerance of ETD [5]. ETD had anti-inflammatory effects with better gastric safety on adjuvant arthritis and normal rats than that of non-selective NSAIDs diclofenac sodium and indomethacin [6]. Compared with other NSAIDs, ETD has shown good efficacy, good gastric tolerance and fewer cardiovascular adverse reactions in various clinical trials, and has minimal hepatic or renal effects [7, 8]. Furthermore, several studies have shown that ETD has anti-proliferative and anti-metastasis effects of cancer [9, 10], while the synovium in RA also has abnormal proliferation, and RA also has metastasis, so we speculated that the ETD might also inhibit the hyperplasia and metastasis of RA.

Data independent acquisition (DIA) mass spectrometry (MS) is one of the most popular mass spectrometry acquisition technologies in recent years, which once led to the new development of quantitative proteomics [11]. Parallel reaction monitoring (PRM) is the gold standard of targeted proteomics. Because no specific antibody is needed, PRM-MS is expected to replace traditional western blot and other verification methods in the future [12]. DIA and PRM showed comparable linearity, accuracy and precision in differential proteome [13]. Here, DIA-based quantitative proteomics was used to explore the differential proteins in synovium between CIA rats and normal rats, and that between CIA rats and ETD treatment rats, and PRM-targeted proteomics was used to validate the results. Based on omics data, the mechanisms hidden behind the protective effects of ETD on RA were further investigated.

## Results

### ETD significantly modified the symptoms of CIA rats (including gross view, arthritis index, joint swelling degree and pain behavior)

Rats in the control group demonstrated no gross signs of arthritis. Immunization with collagen was associated with expected increases in right heel width, and the swelling degree reached its peak at 30 days after immunization. After that, the swelling degree decreased slightly, but it was still obvious. The secondary swelling appeared around the 10th day after immunization, showing swelling of contralateral foot and forefoot, the total score reached its peak around 2 months after immunization, since then, the right hind feet were significantly swollen and deformed, and the secondary lesions of contralateral foot were serious in vehicle- CIA rats throughout the study. Immunization with collagen was associated with increased pain behavior measured as reduced paw withdrawal thresholds. The pain threshold decreased sharply immediately after immunization, and it elevated slightly with time, but still significantly lower than that of the normal control group.

**Fig. 1:**
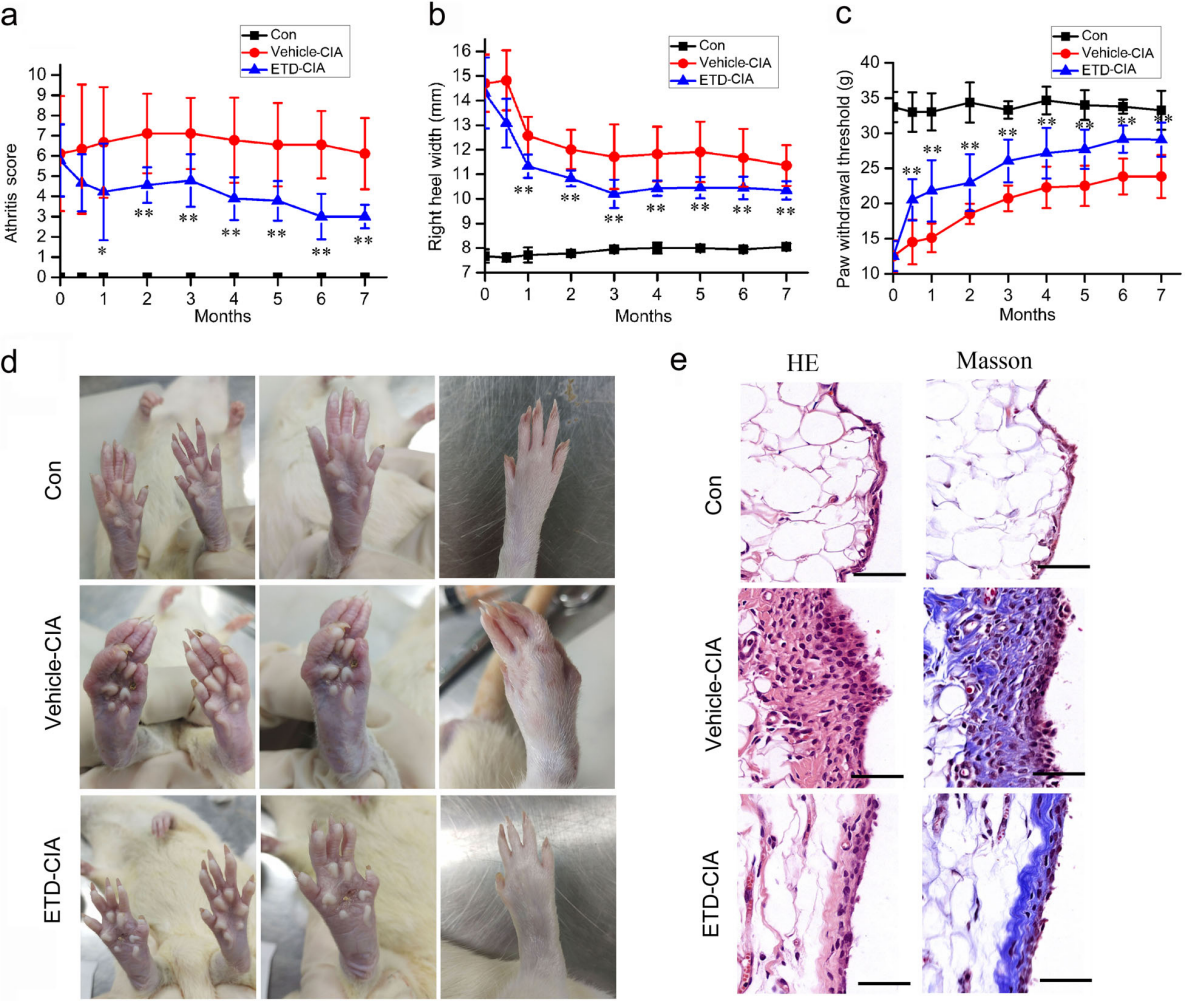
Effects of ETD on CIA rats. Vehicle-CIA rats had obvious symptoms of arthritis measured as increased total scores (**a**) and the right heel width (**b**) through 7 months after collagen injection compared with saline-injected controls, and the paw withdrawal thresholds was significantly decreased (**c**). The total scores and right heel width were significantly decreased, the paw withdrawal thresholds were significantly increased in ETD-CIA rats, **p < 0.01 compared with Vehicle-CIA rats (n = 8). **d** The appearance of both hind limbs 7 months after modeling, the right hind foot was significantly swollen and deformed, the secondary lesions of contralateral foot were serious in vehicle-treated group, while in ETD-CIA rats, the swelling and deformation of the feet were mild (n = 8). **e** Representative micrographs depicting HE stained and Masson synovial sections observed under 40 × objectives. Scale bars, 50 μm. The synovial histopathology of control group showed the normal structure of the synovial lining with clear and smooth features, without hyperplasia. The rats in the vehicle-treated model group showed infiltration of plasma cells, macrophages and lymphocytes as well as proliferative synovial cells, and synovial lining showed significant thickening and positive staining of Masson. The lesions in ETD-CIA rats were alleviated significantly. C: Control rats; V: Vehicle-CIA rats; E: ETD-CIA rats

Two weeks after immunization, ETD was given to rats daily as a single dose by gavage for 7 months. Since half a month after administration ETD, the symptoms were relieved, the right heel width and total score were significantly decreased and the pain threshold was significantly increased, and the symptoms were significantly relieved during the whole 7 months (Fig. 1a, b and c). Seven months after immunization, the swelling and deformation of the feet were mild in the ETD treatment group (Fig. 1d).

**Fig. 2:**
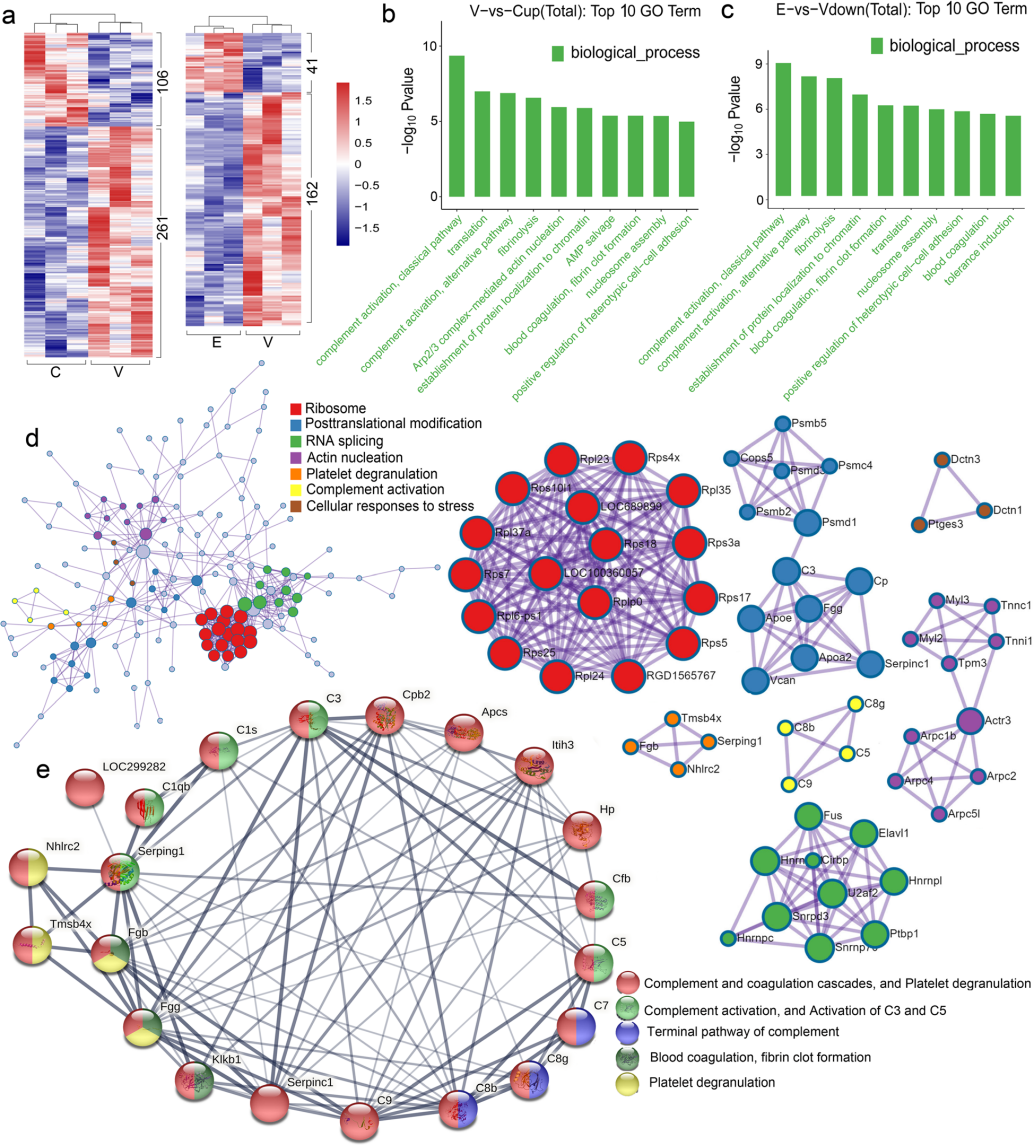
Visualizations of Go functional enrichment and interactome analysis results. **a** The cluster heat map of 367 differentially expressed proteins between Vehicle-CIA rats and Control rats. Among them, 261 upregulated ones and 106 downregulated ones. The cluster heat map of 203 differentially expressed proteins between ETD-CIA rats and Vehicle-CIA rats. Among them, 41 upregulated ones and 162 downregulated ones. Red is upregulated protein; blue is downregulated protein. The clustering is based on the log standard abundance of the significant differential expressed proteins (*P* value<0.05). **b** GO enrichment analysis of the top10 bar chart of 261 upregulated proteins in Vehicle-CIA rats compared with Control rats. **c** GO enrichment analysis of the top10 bar chart of 162 downregulated proteins in ETD-CIA rats compared with Vehicle-CIA rats. C: Control group; V: Vehicle-CIA rats; E: ETD-CIA rats. **d** Metascape visualization of the interactome network formed by 245 with gene names in 261 up-regulated proteins where the Molecular Complex Detection MCODE complexes are colored according to their identities. Nine MCODE complexes automatically identified in Metascape, colored by their identities. **e** The complement and coagulation cascades, and platelet degranulation cluster abstained by STRING online analysis. The local network clusters were colored by different colors

### ETD significantly alleviated the pathological changes of synovium in CIA rats

At the end of experiment, the pathological examination of synovial tissues was carried and analyzed. The normal synovial lining is smooth and consists of two to three layers of synovial cells. The proliferative synovial lining showed infiltration of plasma cells, macrophages and lymphocytes as well as proliferative synovial cells. Chronic inflammation leads to neovascularization and fibrous tissue proliferation, which all cause irregular synovial thickening. Masson trichrome staining is a method to identify collagen. Hyperplastic synovial lining showed positive staining of Masson (Fig. 1e). Synovial hyperplasia and inflammatory cells infiltrating were severe in vehicle treated group, and they were significantly alleviated in ETD treatment group (Fig. 1e). Each components score (inflammatory cell infiltration, synovial cells proliferation, neovascularization and fibrous tissue proliferation) and total score total synovitis score (TSS) were significantly increased in vehicle-treated model group, and they were significantly decreased in ETD treatment group (Table 1).

**Table 1:** Components and total synovitis score

	Con	Vehicle-CIA	ETD-CIA
Inflammatory cell infiltration	0.6 ± 0.5	3.4 ± 0.7^##^	1.4 ± 0.5^**^
synovial cells proliferation	0.2 ± 0.5	3.3 ± 0.9^##^	1.2 ± 0.4^**^
neovascularization	0.1 ± 0.4	2.1 ± 0.8^##^	0.9 ± 0.6^**^
fibrous tissue proliferation	0.6 ± 0.5	3.7 ± 0.5^##^	1.4 ± 0.5^**^
Total synovitis score	1.6 ± 0.9	12.6 ± 2.3^##^	3.6 ± 1.0^**^

Data are expressed as mean ± SD (*n* = 5), ^##^*p* < 0.01 compared with control group; ^**^p < 0.01 compared with Vehicle-CIA group

### DIA-based proteomics analysis

A total of 4934 proteins were identified in this study (Supplementary Table S1), among which 2463 proteins were quantitated (Supplementary Table S2). The proteomic analysis highlighted 367 differentially expressed proteins, including 261 upregulated ones and 106 downregulated ones between vehicle-treated group and the control group; 203 differentially expressed proteins between vehicle-treated group and ETD-treated group, among them, 41 were upregulated and 162 were downregulated in ETD-treated group. The Sketch heat maps of cluster analysis of difference comparison groups were shown in Fig. 2a. Detailed heat map was shown in supplementary Fig. 1.

### Bioinformatics analysis

Two hundred sixty-one upregulated proteins in the vehicle treated model group were analyzed using GO enrichment analysis. Screening the three categories of GO entries with the number of corresponding proteins greater than 1. According to the - log10 *p* value of each entry, 10 entries are sorted from large to small. GO enrichment analysis of the top10 bar chart showed as Fig. 2. Among the upregulated proteins in vehicle treatment group, the top 10 terms of upregulated proteins in biological process category were “complement activation, classical pathway”, “translation”, “complement activation, alternative pathway”, “fibrinolysis”, “Arp2/3 complex-mediated actin nucleation”, “establishment of protein localization to chromatin”, “AMP salvage”, “blood coagulation”, “fibrin clot formation”’ “nucleosome assembly” (Fig. 2b). Most of these terms were downregulated in ETD treatment group (Fig. 2c).

Protein-protein interaction enrichment analysis was analyzed by Metascape online analysis (https://metascape.org/) and STRING online analysis (https://string-db.org/). Functional links between proteins can usually be inferred from the genomic correlation between the genes that encode them. Two hundred sixty-one upregulated proteins in vehicle-treated model group were input, 245 of which were used for analysis. The interaction network diagram and cluster analysis are shown in Fig. 2d. According to the classification of Metascape, the differential proteins clustered in the following categories were highlighted: “Ribosome”. “Posttranslational modification”, “RNA splicing”, “Actin nucleation”, “Platelet degranulation”, “Complement activation” and “Cellular response to stress”. STRING online analysis results highlighted the cluster of complement and coagulation cascades, and platelet degranulation, and the proteins in this cluster was classified and marked with different color according to their properties (Fig. 2e).

**Fig. 3:**
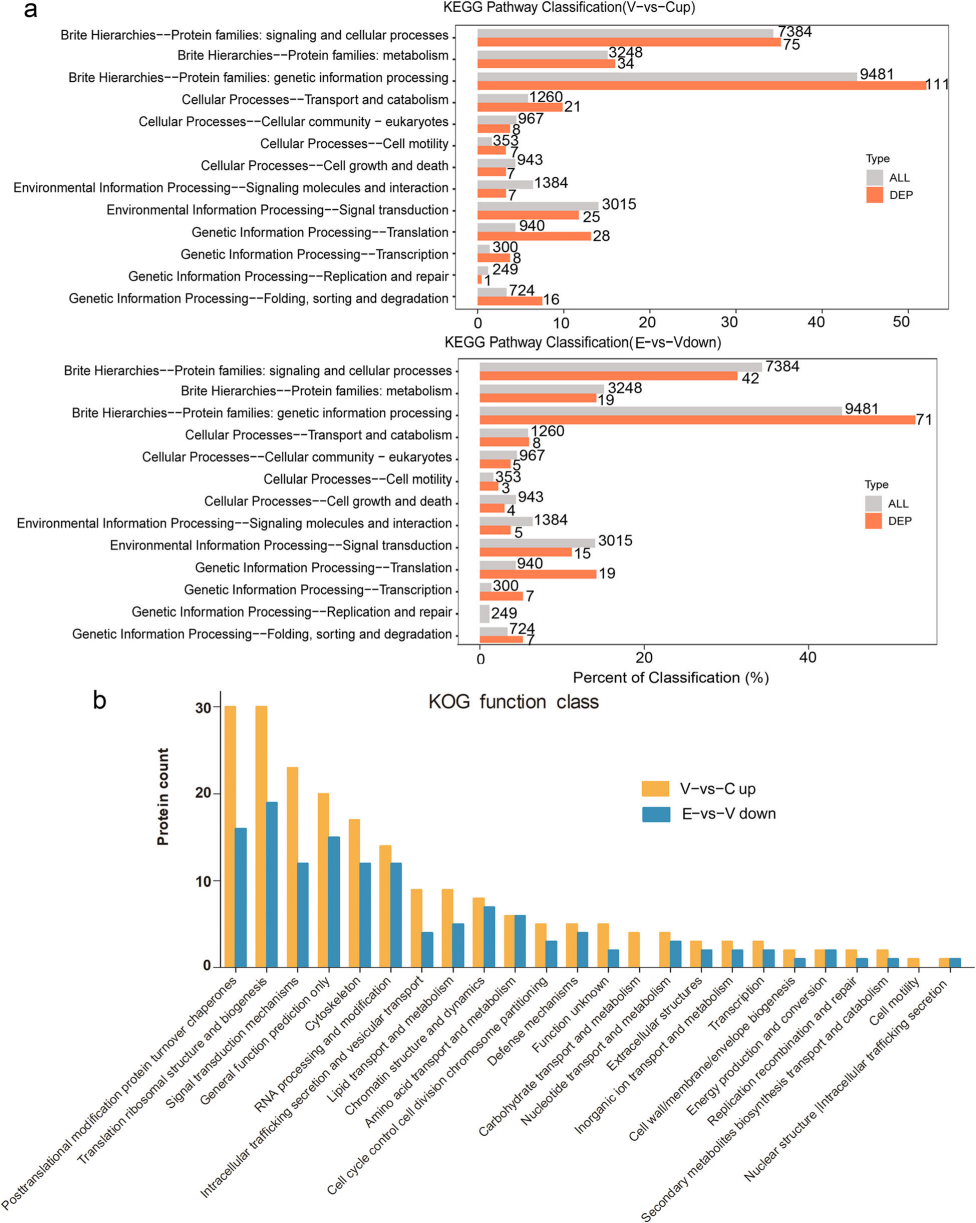
Visualizations of KEGG pathway classification and KOG function class analysis results. **a** Comparison of the distribution of differentially expressed proteins (DEP) and all proteins (ALL) at KEGG Level 2. The pathway to enrich the largest number of proteins is genetic information processing pathway. There are 111 proteins in upregulated proteins in Vehicle-CIA rats compared with control group, and 71 ones in downregulated proteins in ETD-CIA rats compared with Vehicle-CIA rats. **b** KOG function class analysis of upregulated proteins in Vehicle-CIA rats and downregulated proteins in ETD-CIA rats. C: Control group; V: Vehicle-CIA rats; E: ETD-CIA rats

KEGG (http://www.genome.jp/kegg/) is a database of biological systems that integrates genomic, chemical and systemic functional information. KEGG provides a reference knowledge base for linking genomes to life through the process of PATHWAY mapping, it is also the main public database for systematic analysis of protein metabolic pathways in cells. KEGG pathway classification results were shown in Fig. 3a. In KEGG Classification level1, the Genetic Information Processing pathway attracted our attention, a total of 111 upregulated proteins were enriched in this pathway, and 71 ones were downregulated in ETD treatment group. This pathway included several level2 pathway, such as “Transcription”, “Translation”, “Replication and repair” and “Folding, sorting and degradation”.

KOG is clusters of orthologous groups for eukaryotic complete genomes, KOG analysis deeply studies the function and evolution pattern of eukaryotes. Two hundred sixty-one upregulated proteins in vehicle-treated model group were input, 210 of which were used for KOG function class analysis, and so that of 132 of 161 upregulated proteins in the ETD treatment group. As shown in Fig. 3b, according to the analysis results, a total of 24 items were listed, including “Posttranslational modification, protein turnover, chaperones”, “Translation, ribosomal structure and biogenesis”, “Signal transduction mechanisms”, “General function prediction only”, “Cytoskeleton”, “RNA processing and modification”, “Intracellular trafficking, secretion, and vesicular transport”, “Lipid transport and metabolism”, “Chromatin structure and dynamics”, “Amino acid transport and metabolism”, “Cell cycle control, cell division, chromosome partitioning”, “Defense mechanisms”, “Function unknown”, “Carbohydrate transport and metabolism”, “Nucleotide transport and metabolism”, “Extracellular structures”, “Inorganic ion transport and metabolism”, “Transcription”, “Cell wall/membrane/envelope biogenesis”, “Energy production and conversion”, “Replication, recombination and repair”, “Secondary metabolites biosynthesis, transport and catabolism”, “Cell motility” and “Nuclear structure Intracellular trafficking, secretion”.

### ETD inhibited synovial inflammation, fibrosis and hyperplasia by downregulating complement and coagulation cascades, and platelet degranulation cluster

**Fig. 4:**
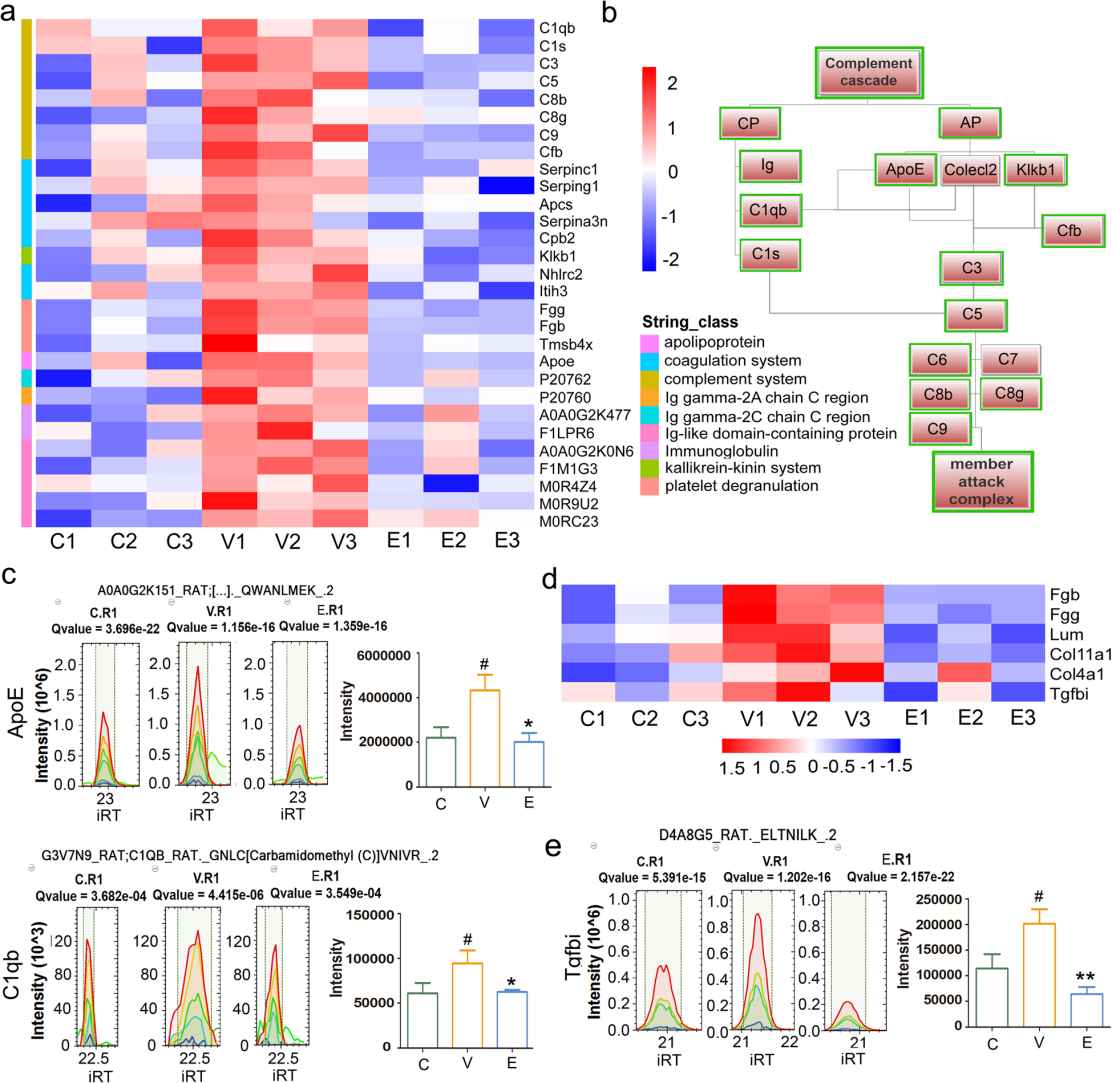
The proteins enriched in cluster of complement and coagulation cascades, and platelet degranulation and their role in RA. **a** The heatmap of the proteins enriched in cluster of complement and coagulation cascades, and platelet degranulation as well as immunoglobulins. **b** Schematic diagram of up-regulated proteins involved in complement system. The upregulation of immunoglobulins, Colecl2, Apoe, Klkb1 as well as a series of complement compartments C1s, C1qb, C3, C5, C7, C8g, C9 represented the activation of complement cascade in present study, and those down-regulated proteins in ETD treatment group were marked with green borders. **c** The expression of ApoE and C1q were verified by PRM, the results were consistent with that of DIA. **d** The upregulation of Fgb, Fgg, Tgfbi, Lum, CIV, CXI contributed to the synovial fibrosis, and ETD reduced synovial fibrosis by downregulating the expressions of Fgb, Fgg, Tgfbi, Lum and CXI. **e** The expression of Tgfbi were verified by PRM, the result was consistent with that of DIA. # *p* < 0.05 compared with Control rats; *p < 0.05, **p < 0.01 compared with Vehicle-CIA rats. C: Control group; V: Vehicle-CIA rats; E: ETD-CIA rats

Combined with the results of Go and Metascape analysis, the complement and coagulation cascades, and platelet degranulation cluster obtained from STRING analysis attracted our attention, with a total of 21 proteins, including Cfb, Serpinc1, Serping1, C8b, Apcs, LOC299282, Cpb2, C1s, C1qb, C9, Klkb1, Nhlrc2, Itih3, C5, Fgg, Hp, Fgb, C7, C8g, Tmsb4x, C3. Except C7, the rest of 20 proteins were down-regulated in ETD treatment group, they were showed in Fig. 4a.

The classical complement pathway activation index reflects the disease activity of rheumatoid arthritis [14]. In addition to complement members, several factors involved complement cascades were found upregulated in present study. A large number of immunoglobulins were upregulated in synovium tissue in CIA rats (Fig. 4a), which represented antibody mediated the classical pathway was activated in present study [15]. As reported, Collectin-12 (Colecl2), Apolipoprotein E (ApoE) and Plasma kallikrein (Klkb1) could trigger complement pathway [16–19], they might also participate complement system activation in present study.

What is noteworthy is that the kallikrein-kinin system might also be involve in RA and may be the target of ETD. As reported, beside the control of prostaglandins by inhibiting COX2, ETD might also inhibit active oxygen generation and bradykinins formation [20]. FXIIa activates plasma prokallikrein (PK) to plasma kallikrein (PKA, Klkb1), which in turn activates FXII and the kallikrein kinin pathway, leading to local increase of bradykinin and further pro-inflammatory effects, such as vascular permeability, vasodilation and immune cell activation [21]. Here, the expression of Klkb1 was significantly upregulated in the synovium tissue of CIA rats, and ETD treatment significantly downregulated it, which indicated that ETD alleviated CIA might also by inhibiting kallikrein-kinin system.

In summary, the upregulation of immunoglobulins, Colecl2, Apoe, Klkb1 as well as a series of complement compartments C1s, C1qb, C3, C5, C7, C8g, C9 represented the activation of complement cascade in present study. As shown in Fig. 4b, a schematic diagram of up-regulated proteins involved in complement system was constructed, in which those down-regulated proteins in ETD treatment group were marked with green borders.

Several components of the coagulation system were also upregulated, such as Fgb, Fgg and Thymosin β4 (Tmsb4x). Fibrinogen (Fg), as a downstream target of thrombin, is one of the most effective contributors of all coagulation proteins to inflammatory response. In addition to strong destructive tissue inflammation, fibrinogen seems to be the driving factor of chronic low-grade inflammation. Fibrinogen-β (Fgb) polymerizes with fibrinogen - α (Fga) and fibrinogen-γ (Fgg) to form insoluble fibrin matrix and fibrin polymerization may be the key mechanism determining the pro-inflammatory ability of Fg. Fibrin is common in inflammatory foci, and extravascular fibrin deposition aggravates inflammation in a series of disease models [22].

Chronic inflammation leads to neovascularization and fibrous tissue proliferation, which all cause irregular synovial thickening. Fibrosis is a sign of excessive accumulation of collagen, which ultimately leads to organ failure [23]. In arthritis, synovial fibrosis might be the main reason of joint stiffness [24]. Here, hyperplastic synovium showed positive staining of Masson (Fig. 1e). The results of omics also showed that Collagen type IV alpha 1 chain (Col4a1), Collagen alpha-1(XI) chain (Col11a1) were upregulated in synovium tissue in CIA rats. Besides, another collagen related protein Lumincan (Lum) was also found to be upregulated. It was also found that one fibrosis factor Transforming growth factor-β-inducible protein (Tgfbi) was upregulated in the synovial tissue of CIA rats. In summary, members of the coagulation system (such as Fgg, Fgb) are related mediators of inflammatory processes, the upregulation of Fgb, Fgg, Tgfbi, Lum, CIV, CXI contributed to the synovial fibrosis, and ETD reduced synovial fibrosis by downregulating the expressions of Fgb, Fgg, Tgfbi, Lum and CXI (Fig. 4d).

It is noteworthy that Tmsb4x has not been reported in RA so far, it might be a new target for RA treatment. Tmsb4x is a natural peptide with low molecular weight, which plays an important role in the repair and regeneration of damaged cells and tissues. After injury, platelets, macrophages and other cells release Tmsb4x to protect cells and tissues from further damage, reduce apoptosis, inflammation and microbial growth. But Tmsb4x also can bind to actin to promote cell migration, including mobilization, migration and differentiation of stem/progenitor cells, forming new blood vessels and regenerating tissues [25]. It could promote tumor proliferation and migration [26]. We suspected that it could also promote RA development and metastasis in the chronic stage of RA. ETD alleviated the syndrome of CIA rats might also through downregulating the expression of Tmsb4x.

### ETD inhibited synovial hyperplasia by downregulating proliferation related proteins

**Fig. 5:**
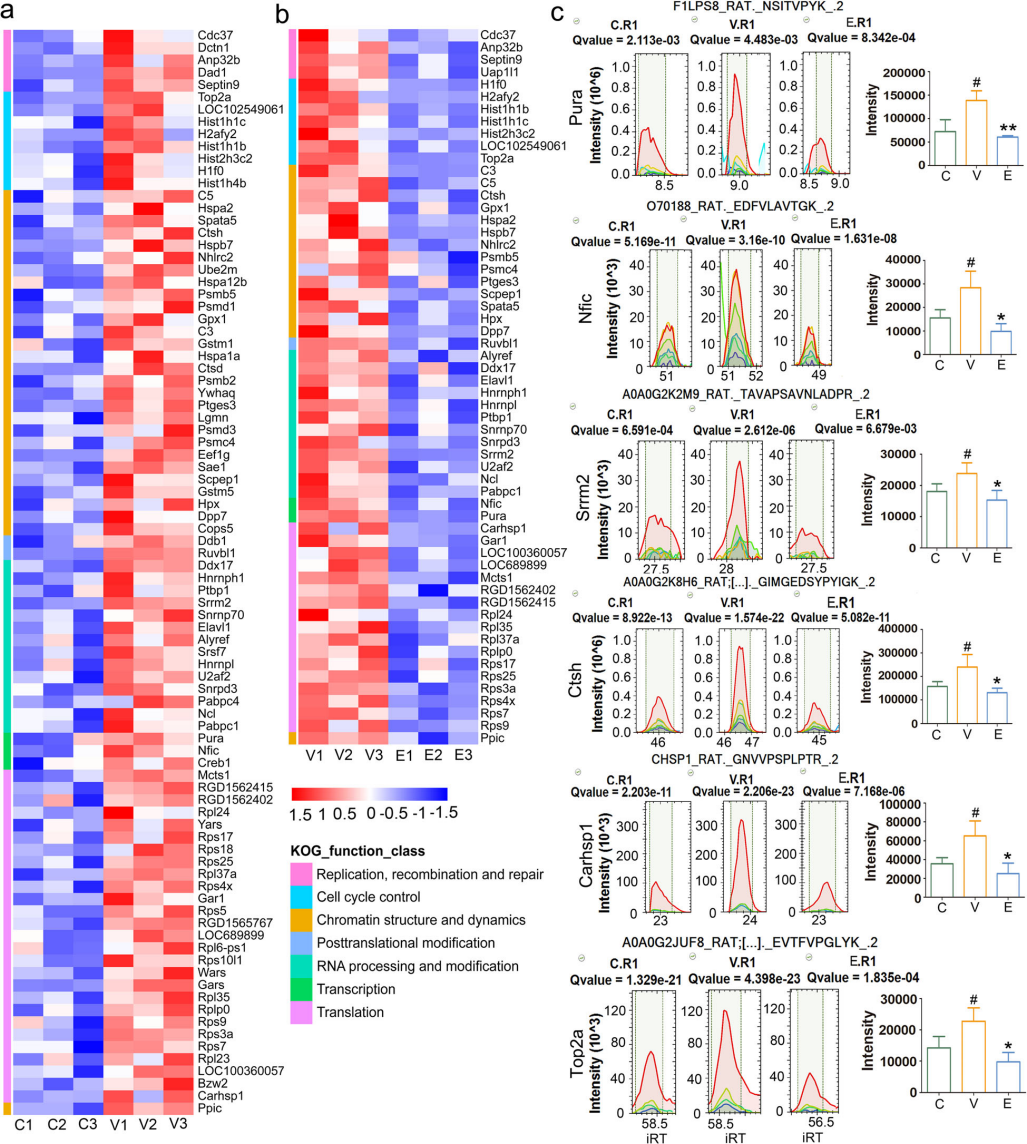
The differential proteins enriched in central dogma. **a** The heap map of 89 upregulated proteins in synovial tissue of vehicle-CIA rats compared with normal control rats. They were classified in the following events according to the KOG function analysis: “Cell cycle control, cell division, chromosome partitioning”, “Chromatin structure and dynamics”, “Transcription”, “Replication, recombination and repair”, “RNA processing and modification”, “Translation, ribosomal structure and biogenesis”, “Posttranslational modification, protein turnover, chaperones”. **b** 59 downregulated ones in ETD-CIA rats compared with that in vehicle- treated rats. **c** The represent proteins in each event of central dogma was selected and verified by PRM method, including Pura, Nfic, Srrm2, Carhsp1 and Top2a, and the results were well consistent with that of DIA, which confirmed the accuracy of DIA proteomics results. # *p* < 0.05 compared with Control rats; *p < 0.05, **p < 0.01 compared with Vehicle-CIA rats. C: Control group; V: Vehicle-CIA rats; E: ETD-CIA rats

Systems biology and its related bioinformatics are driving our understanding of central dogma and cell biology [27]. The central dogma refers to the transmission of genetic information from DNA to RNA, and then from RNA to protein, it includes several events, such as transcription, RNA editing, splicing, translation, posttranslational modification and DNA replication. The central dogma occurs in the reproductive process or cell proliferation, so the overexpression of central dogma related proteins might represent the prosperous proliferative activity. As shown in Fig. 3, compared to KEGG pathway classification, KOG functional analysis revealed the central dogma events more comprehensively. It showed that the 89 of 261 upregulated proteins were distributed in all events of genetic central dogma and its extension, including “Transcription”, “Chromatin structure and dynamics”, “Replication, recombination and repair”, “Cell cycle control, cell division, chromosome partitioning”, “RNA processing and modification”, “Translation, ribosomal structure and biogenesis”, “Posttranslational modification, protein turnover, chaperones”. They are shown in Fig. 5a and listed by their gene names. These proteins reflected the active proliferation of synovial tissue, for many of them were found involved in the abnormal proliferation of cancer cells. We carried a literature research on them, and their RA or cancer related observations were summarized in Table 2. Among these 89 up-regulated proteins, 59 were downregulated in ETD treatment group (Fig. 5b), which indicated the inhibition of the active proliferation of synovial tissue.

**Table 2:** Upregulated proliferation related proteins in RA related observations and cancer-related observations in synovial tissue of CIA rats

Protein description	RA related observations	Cancer related observations
**DNA processing and modification**		
Cyclic AMP-responsive element-binding protein 1 (Creb1)	Participated in in the movement, survival, proliferation and chemokine/ cytokine synthesis of fibroblast like synoviocytes [28]	Involve in the migration and proliferation of cancer cells [29]
Transcriptional activator protein Pur-α (Pura) ^a^	–	Assist DNA repair after DNA damage [30]
Nuclear factor I-C (Nfic)^a^	–	Promote the transcription of lncRNA LBX2-AS1 in gastric cancer (GC) cells [31]
RuvB-like 1(Ruvbl1, Tip49) ^a^	–	Modulated wnt/β-catenin activation [32]
Histones	Participated in a variety of autoimmune diseases including arthritis [33]	Histone modifications are the targets of epigenetic drugs against cancer [34]
Topoisomerase IIα (Top2a) ^a^	Anti-Topo II α antibodies were detected in rheumatoid arthritis [35]	The abnormal changes of Top2a and its interacting proteins and their modifications may play a key role in human cancer [36]
**RNA processing and modification**		
Small nuclear ribonucleoprotein Sm D3 (Snrpd3, SmD3) ^a^	–	As inhibitor of p53 activity in multiple NSCLC cell lines [37]
U1 small nuclear ribonucleoprotein 70 kDa (Snrnp70, U1–70K) ^a^	U1–70K is the target of self-reactive B cells and T cells in several rheumatic diseases [38]	With robust and consistent expression levels across breast cancers [39]
U2 snRNP auxiliary factor large subunit (U2af2) ^a^	–	Promote the Warburg effect and tumorigenesis [40]
Serine/arginine repetitive matrix protein 2 (Srrm2, SRm300) ^a^	–	Silence Srrm2-AS can block cell cycle and promote cell apoptosis [41]
RCG61762, isoform CRA_d (Srsf7)	–	SRSF7 knockdown promotes apoptosis of colon and lung cancer cells [42]
Embryonic Lethal Abnormal Vision-Like Protein 1 (Elavl1, HuR) ^a^	–	Enhance cell proliferation and survival, promote angiogenesis, evading the recognition of the immune system, and increase the invasion and metastasis potential of cancer cells [43]
Polypyrimidine tract-binding proteins 1 (Ptbp1) ^a^	Essential for B cell ontogeny and B cell receptor (BCR)-mediated antibody production [44, 45]	Promote the proliferation and metastasis of cancer [46]
Heterogeneous nuclear ribonucleoprotein H (Hnrnph1) ^a^	–	Increased expression of hnrnph1 is associated with poor prognosis of Mantle cell lymphoma [47]
Heterogeneous nuclear ribonucleoprotein L (HnrnpL)^a^	Involved in the regulation of TNF α gene transcription [48]	As a prostate cancer dependency regulating RNA splicing [49]
**Translation, ribosomal structure and biogenesis**		
Tryptophanyl-tRNA synthetase (Wars, TRPRS)	–	Possible contributor during uveal melanoma progression [50]
Tyrosyl- tRNA synthetase (Yars)	–	Promotes gastric cancer progression through activating PI3K-Akt signaling [51]
Basic leucine zipper and W2 domains 2 (Bzw2)	–	Promote the malignant progression of CRC (Colorectal cancer) via activating ERK/MAPK signaling [52]
Multiple copies in T-cell malignancy 1 (Mcts1) ^a^	–	Promoting cell proliferation, inhibiting apoptosis and promoting angiogenesis [53]
Eukaryotic initiation factor 4B (Eif4b)	–	The depletion of eIF4B in cancer cells can reduce cell proliferation [54]
Ribosomal proteins^a^	Ribosomal proteins were upregulated in the synovial membranes of female patients with Osteoarthritis [55]	Cope with increased protein synthesis and maintain unrestricted growth of cancer cell [56]
Calcium-regulated heat stable protein 1 (Carhsp1)	Regulates the stability of TNF -α mRNA [57]	Phosphorylation of CARHSP1^S30,S32^ could be potential candidates associated with K8 phosphorylation mediated tumorigenicity [58]
**Posttranslational modification, protein turnover, chaperones**		
cathepsin D (Ctsd)	Contributed to the pathogenesis of RA [59]	Ctsd is a tumor-specific extracellular target in TNBC Triple-negative breast cancer (TNBC) [60]
cathepsin H (Ctsh) ^a^	–	Promoted cancer cell progression by affecting integrin activation and adhesion strength [61]
Lysosome-associated membrane glycoprotein 1 (Lamp1)	–	Supported tumor growth and metastasis [62]
Dipeptidyl peptidase 2 (Dpp7) ^a^	–	Highly expressed in Multiple myeloma [63]
Proteasome^a^	Proteasome inhibitors (PIs) can inhibit the activation of nuclear factor (NF)-κB and transcriptional regulation of pro-inflammatory cytokine release, and/or (b) induce apoptosis of activated immune cells [64]	A multifaceted target for anti-cancer therapies [65]
Glutathione peroxidase (Gpx1)	Aberrant expression of Gpx contributed to the etiology of rheumatoid arthritis (RA) [66]	Gpx1 is commonly overexpressed in most human cancers and can be used as a prognostic biomarker for identifying cancer types [67]
HSP70^a^	Induce Th17 frequency and Th17 /Treg ratio in vivo, reflecting its pro-inflammatory activity [68]	Promoted tumorigenesis in many kinds of cancer [69]
Heat shock protein 27 (Hspb7) ^a^	Significantly increased in RA synovial tissues [70]	Protected cancer cells from apoptosis inducing agents [71]
Prostaglandin E synthase 3 (Ptges3, p23) ^a^	–	Beneficial to the abnormal proliferation of cancer cells [72]
**Cell cycle control, cell division, chromosome partitioning**		
Cell division cycle 37 (Cdc37) ^a^	–	Promoting cancer cell proliferation, G1-S conversion and inhibiting apoptosis [73]
Cytoskeleton interacting protein septin9 (Septin9) ^a^	–	Has a carcinogenic effect and is an oncogene [74]
		Promotion of migration and invasion of cancer cells [75]

^a^: Downregulated in ETD-CIA group; −: No retrieved

### PRM proteomics validation

We selected some represent proteins in the terms of complement activation and fibrosis and verified them by PRM method. The expression of ApoE, C1q and Tgfbi were consistent with that of DIA (Fig. 4c and e). According to KOG function class analysis, the represent proteins in each event of central dogma were verified by PRM method, including Pura, Nfic, Srrm2, Carhsp1 and Top2a, the results were well consistent with that of DIA (Fig. 5c), which the confirmed the accuracy of DIA proteomics results.

### The safety of ETD treatment

The safety of 7 months treatment of ETD was investigated by blood biochemistry analysis. Compared with normal control rats and vehicle-CIA rats, no obvious abnormality was found in biochemical tests in ETD-CIA rats. It indicated that long term administration of ETD for 7 months had no toxic reactions such as liver and kidney, which further proved the safety of ETD. The results were listed in Table 3.

**Table 3:** The results of blood biochemical tests

		Con	Vehicle-CIA	ETD-CIA
Glu-G	mmol/L	10.72 ± 1.25	11.74 ± 6.43	9.43 ± 4.23
T-Bil-V	μmol/L	0.58 ± 0.21	0.19 ± 0.40	0.21 ± 0.27
ALT	U/L	49.25 ± 3.90	57.90 ± 18.78	63.56 ± 36.47
AST	U/L	133.68 ± 22.53	128.67 ± 26.01	120.19 ± 56.61
ALP	U/L	97.75 ± 26.75	153.28 ± 93.99	97.90 ± 41.24
γ-GT	U/L	0.90 ± 0.36	1.24 ± 0.88	1.00 ± 0.44
TP	g/L	53.43 ± 2.11	56.94 ± 3.44	55.43 ± 19.68
TG	mmol/L	0.75 ± 0.19	0.63 ± 0.16	0.72 ± 0.28
LDL-C	mmol/L	0.67 ± 0.04	0.59 ± 0.11	0.68 ± 0.24
HDL-C	mmol/L	1.85 ± 0.13	1.75 ± 0.26	1.78 ± 0.59
TC	mmol/L	2.39 ± 0.17	2.28 ± 0.28	2.42 ± 0.83
UREA	mmol/L	5.97 ± 0.35	6.36 ± 1.07	6.69 ± 2.31
CK-MB	U/L	860.65 ± 406.12	711.17 ± 457.68	738.49 ± 401.94
CK	U/L	430.93 ± 175.77	457.21 ± 215.77	382.81 ± 202.93
LDH	U/L	1099.15 ± 481.02	877.71 ± 532.64	909.40 ± 459.93
ALB II	g/L	29.10 ± 1.38	29.40 ± 0.80	29.43 ± 10.38
CREA-S	μmol/L	35.98 ± 2.65	34.76 ± 5.29	34.07 ± 13.54

Data are expressed as mean ± SD (*n* = 8); there were no significant differences among three groups

## Discussion

It is well known that ETD can reduce pain and inflammation by inhibiting COX2, and it has pain and joint swelling relief effects on RA [8, 76, 77]. But it is generally believed that NSAIDs do not have the same therapeutic effect as DMARDs. Our study provided evidence that ETD also has disease modifying anti rheumatic effect. It could reduce primary and secondary lesions of CIA rats by inhibiting synovial inflammation, fibrosis and hyperplasia. Moreover, with the help of proteomic technology and bioinformatics analysis, the molecular mechanisms of synovial hyperplasia in RA were explored, and the mechanisms hidden behind the protective effects of ETD on RA were further investigated.

Our study for the first time to identified so many differential proteins in the synovium of CIA rats, we focused on the upregulated proteins and tried to clarify their roles in RA. STRING analysis highlighted the cluster of complement and coagulation cascades, and platelet degranulation, it involved in synovial inflammation and fibrosis. ETD treatment downregulated most of proteins in this cluster. Our study was consistent with previous research views that the activation of classical pathway (CP) and alternative pathway (AP) are involved in RA [78]. As reported, the main component of Rheumatoid factor (RF) is IgM, including IgG, IgA and IgE. RF-IgG complex in synovium can fix and activate complement, produce C3a and C5a, and attract neutrophils and monocytes to secrete. Neutrophils, monocytes and synoviocytes (type A cells) phagocytize immune complexes, then activate and release lysosomal enzymes, including neutral protease, collagenase and various mediators, such as prostaglandins, leukotrienes and IL-1, leading to the destruction of synovium and articular cartilage [79]. A large number of immunoglobins were found upregulated, they activated CP. Some other factors of complement activation were also found in present study, for example, the upregulations of AopE, Colecl2, Klkb1 also participated the activation of complement system in RA.

The activation of the immune system has been increasingly considered to be related to the procoagulant state of patients with RA. Many different components of the hemostatic system (such as thrombin, fibrinogen, coagulation factor XIII and fibrinolytic system factor) are related mediators of inflammatory process and inflammation control [80]. Furthermore, fibrinogen promoted renal fibrosis by inducing fibroblast proliferation and activating TGF-β1/pSMAD2 signaling [81]. Here, the upregulations of components of the hemostatic system (Fgg, Fgb) participated in inflammation, the upregulation of Fgb, Fgg, Tgfbi, Lum, CIV, CXI contributed to the synovial fibrosis.

As reported, the immune complement system was involved in mucinous cystic neoplasm (MCN), combined with their performance in this study, it supported the inference that the similarity between cancer and RA [82]. Moreover, with the help of KOG analysis, this inference got more supported. According to KOG analysis, the active proliferation of synovial tissue was also reflected by the 89 upregulated proliferation related proteins, As shown in Fig. 5, these proteins were classified according to their roles in central dogma. Among these 89 up-regulated proteins, 59 ones were downregulated in ETD treatment group, which indicated that ETD alleviated the symptom of RA by reducing these proliferation related proteins. We conducted a literature survey for them, and their RA and cancer related observations were listed in Table 2, and classified them by their role in the events of central dogma.

Transcription and replication are two important processes of cell survival and proliferation. Although these two processes are two different events, they are inextricably linked because they share certain proteins [83]. So, several proteins involved in “Transcription”, “Chromatin structure and dynamics”, and “Replication, recombination and repair” were integrated into DNA processes and modifications category in Table 2, including Creb1, Nfic, Pura, Ddb1, Ruvbl1, histones and Top2a. Their upregulations indicated that gene transcription and replication were significantly promoted in the synovium of CIA rats. They might be the main factors to ensure a high level of transcription and replication, and the way to break or inhibit them may be effective strategy for RA.

Transcription control is the first step in the formation of immune response. RNA editing, localization, stability and translation are other key steps in the final gene expression after transcription. The human genome consists of about 20,000 genes encoding proteins, but the transcriptome and proteome are much larger. This result is due to RNA splicing, which converts precocious mRNA into mature protein encoded mRNA by removing intron sequence. RNA splicing is a dynamic reaction catalyzed by a ribonucleoprotein complex called spliceosome, which involves the sequential recruitment and release of many different proteins in pre mRNA transcripts. RNA splicing also has regulatory mechanisms, and if something goes wrong, diseases like cancer or autoimmune diseases can occur [84]. The analysis based on KOG function class showed that 14 proteins upregulated proteins were involved in the category of RNA processing and modification. Among them, Snrpd3, Snrnp70, U2af2, Alyref, Srsf7 and Srrm2 are components of the RNA splicing machinery. RNA binding proteins (RBPs) play regulatory role in post transcriptional processes by acting on regulatory sequences, resulting in differences in mRNA abundance and protein expression of cytokines [85]. In present study, the upregulation of RBPs included HuR (Elavl1), Ptbp1. Among14 upregulated RNA related proteins in synovial tissue of CIA model, 12 of them were downregulated in ETD treatment group, they are Alyref, Ddx17, Elavl1, Hnrnph1, Hnrnpl, Ptbp1, Snrnp70, Snrpd3, Srrm2, U2af2, Ncl, and Pabpc1. Furthermore, as shown in Table 3, according to literature research, most of above RNA related proteins played roles in the occurrence and development of tumors. Some tumors are highly dependent on splicing function to maintain survival, which makes RNA related events become target for cancer treatment [86]. Among these proteins, U1–70K (Snrnp70) is the target of self-reactive B cells and T cells in several rheumatic diseases [38], Ptbp1 is essential for B cell ontogeny and B cell receptor (BCR)-mediated antibody production [44, 45], HnrnpL involved in the regulation of TNF α gene transcription [48]. Only these three proteins might be associated with RA, except this, there’s nothing more evidences about these RNA processing and modification proteins are related with RA. We proposed the rest of RNA-related proteins might also be involved in the synovial hyperplasia, and they might be new targets of RA treatment.

According to the KOG function class analysis, 28 up-regulated proteins were specifically related to translation, ribosomal structure and biogenesis, including Gars, Wars, Yars, Gar1, Bzw2, Carhsp1, Mct-1, Eif4b, 40S ribosomal protein (S3a, S4, S5, S7, S9, S17, S25, S26) and 60S ribosomal proteins (L22, L23, L23a, L24, L27a, L35, L37a, L6). They also related with cancer progression, and their cancer relevant observations were showed in Table 2. They might not only cope with increased protein synthesis and maintain unrestricted growth of cancer, but also account for the increased protein synthesis in synovial proliferation in CIA rats. Among them, Ribosomal proteins were upregulated in the synovial membranes of female patients with Osteoarthritis [55], Carhsp1 can regulate the stability of TNF -α mRNA [57], they might be associated with RA, no other proteins, such as Wars, Yars, Bzw2, Mcts1 and Eif4b were reported in RA. 17 of 28 up-regulated proteins were downregulated in ETD-CIA rats indicated protein synthesis was inhibited by ETD treatment.

After protein translation, further posttranslational modification, protein turnover and chaperones are needed to ensure protein function. According to the functional analysis of KOG, 30 related proteins are up regulated, including lysosomal enzymes Ctsd, Ctsh, Lamp1 and Dpp7, proteasome related proteins Psmb2, Psmb5, Psmc4, Psmd1 and Psmd3, protein turnover and chaperones Hspa12b, Hspa1a, Hspa2, Hsp27, Ptges3 and so on. Lysosomal cathepsins play an important role in the induction and diagnosis of RA. The levels of several tissue proteases (B, D, G, K, l and S) in serum and synovial fluid of patients are considered as the basis of RA diagnosis, regulating cathepsin activity can improve the pathological characteristics of some autoimmune and inflammatory diseases [87]. A study showed that the activities of cysteine protease Ctsb, Dpp1, aspartic protease Ctsd and two glycosidases are increased in all patients, which is related to the progress of RA [88]. Several cathepsin inhibitors (B, D, l, K and S) have been described and their activity in rheumatic autoimmune diseases has been evaluated [89]. In present study, 16 lysosomal enzymes (Ctsa, Ctsb, Ctsc, Ctsd, Ctsh, Ctss, Dpp3, Dpp4, Dpp7, Acp2, Scarb2, Lamp1, Lamp2, Lyz2, Tpp1, and Tpp2) were identified and quantified (Supplementary Table 2), among them, Ctsd, Ctsh, Lamp1 and Dpp7 were significantly upregulated in CIA rats, and Ctsh, Dpp7 were significantly downregulated by ETD treatment. Although many lysosomal enzymes were reported in RA, but Ctsh and Dpp7 were not yet found. As shown in Table 2, Ctsd and Ctsh was reported to promote cancer cell progression [60, 61].

The 26S proteasome plays a key role in maintaining protein homeostasis by removing misfolded or damaged proteins that may impair cell function, and removing proteins that are no longer needed. This type of proteolysis is necessary in many ways, including spermatogenesis or generation of a subset of MHC class I-presented antigenic peptides. Many diseases, such as neurodegenerative diseases and cancer are closely related to the activity of proteasome, which makes it an important pharmacological target [65]. The 26S proteasome is composed of two 19S regulatory particles and 20S core proteasome complex. Within the 20S core complex, Psmb5 displays a chymotrypsin-like activity. Bortezomib is the first-class proteasome specific drug approved by FDA, which can reversely inhibit chymotrypsin activity of 20S proteasome [90]. The drug has been approved for the treatment of multiple myeloma, recurrent/refractory multiple myeloma and mantle cell lymphoma. In addition, it can also be used in autoimmune diseases such as RA. In present study, Psmb2, Psmb5, Psmc4, Psmd1 and Psmd3 were up-regulated in CIA rats, and Psmb5 and Psmc4 were significantly decreased in ETD treatment group, which indicated the activity of proteasome was decreased by ETD treatment.

Hsp70, Hsp40, Hsc70, Cdc37, p23 (Ptges3) and immunophilins are different cochaperones of Hsp90, which indispensable in achieving different functions and selectivity through direct protein-protein interactions with Hsp90 [91]. In present study, 13 PPIases were identified (Supplementary Table S2), among them Ppic, Fkbp7 and Fkbp9 were upregulated in CIA rats, all of them were significantly downregulated in ETD treatment group. It is well known that Hsp27, Hsp70, Hsp90 and other chaperone proteins are often enhanced in the process of tumorigenesis, and play an important role in many key steps of tumorigenesis and development [92, 93]. In present study, Heat shock protein family A (Hsp70) member 12B (Hspa12b), Heat shock 70 kDa protein 1A (Hspa1a), Heat shock-related 70 kDa protein 2 (Hspa2) were found upregulated, they belong to molecular chaperones Hsp70/Hsc70, Hsp70 superfamily. Hsp27 (Hspb7) and the cochaperones of Hsp90 (Ptges3, PPIases) were also upregulated, Hspa2, Hspb7, Ppic and Ptges3 were downregulated in ETD treatment group. So far, there are some conflicting conclusions about the role of Hsp70 in RA. Some studies think that it has pro-inflammatory effect, and other think it has anti-inflammatory effect [68]. We think that the role of Hsp70 may be different from the stage of RA, but in the chronic stage, such as this study, the role of Hsp70 should be to promote proliferation, just like its role in tumor. The upregulations Hsp27, Hsp70, and Hsp90 cochaperones might all contribute to synovial hyperplasia, which was another evidence for synovial hyperplasia with tumor proliferation characteristics.

The above series of activities and associated proteins are preparing for cell proliferation. Following these events, cells will enter into the stage of division and proliferation. In present study, the final result of cell proliferation is synovial hyperplasia. There are also some cell division related proteins in the upregulated protein. Cdc37 (p50) and Septin9 are two belong to the catalog of cell cycle control, cell division, chromosome partitioning. They were both involved in tumorigenesis and invasion, and their cancer relevant observations were also listed in Table 2. It should be noted that Septin9 has a carcinogenic effect and is an oncogene an oncogene, which might be associated with RA related cancer. The expression of Cdc37 and Septin9 were both downregulated by ETD treatment.

As reported, the gradual formation of hypoxia and malnutrition microenvironment in RA joints are similar to that of solid tumors [94]. Some markers of RA associated with malignancies have been identified [95]. Here, we provided the evidence at the molecular level that the process of cell proliferation was also very similar between cancer and RA. In other words, tumor and arthritis might have common therapeutic targets, and proteasome inhibitor is a good example [90]. As shown in Table 3, the literature research showed that most of these proteins are related to the abnormal proliferation and metastasis of tumor cells, but few of them have been studied in RA, several proteins were firstly reported associated with RA, such as Pura, Nfic, Ruvbl1, Snrpd3, U2af2, Srrm2, Srsf7, Elavl1, Hnrnph1, Wars, Yars, Bzw2, Mcts1, Eif4b, Ctsh, Lamp1, Dpp7, Ptges3, Cdc37 and Septin9. They may be potential targets for RA, but still need a lot of validation work. The downregulation of these proteins was beneficial for the remission of RA, which was confirmed in ETD treatment group.

As a selective COX-2 inhibitor, more and more studies have shown that ETD has other functions besides analgesia and anti-inflammatory. The overexpression of COX2 was also involved in tumor regeneration, and ETD showed anti-proliferative effects by suppressing COX-2 in cancer [9, 10]. Due to genetic susceptibility, persistence of long-term disease activity under persistent immune stimulation and anti-RA therapy, especially the use of DMARDs, the risk of certain cancers in RA patients would increase, such as lung cancer, lymphoma, non-melanoma skin cancer, and so on [96–98]. As shown in Table 2, so many upregulated proteins in CIA rats in present study were related with cancer, most of them can promote cancer cell proliferation and metastasis, some even have carcinogenic effects. This might be the mechanism of the occurrence of RA related cancer. And their downregulation in ETD treatment group also provided the clue that ETD might also have the potential of preventing tumor occurrence in patients with RA. We hypothesized that ETD alone or combined with DMARDs in the treatment of RA may reduce the risk of cancer, but evidence is still needed to confirm this.

We also investigated the safety of long-term administration ETD. Biochemical results showed that there was no abnormal biochemical index after taking ETD for 7 months, which further validated the safety of ETD.

In conclusion, using DIA and PRM proteomics to study the mechanism of synovial proliferation provided more clues for the anti-synovial hyperplasia therapy. ETD can not only relieve pain and inflammatory but also has disease modifying effect in CIA rats by inhibiting synovial inflammation, fibrosis and proliferation. Long-term use of ETD in RA patients has a good efficacy and safety, it still has important clinical value.

## Materials and methods

### Animals and reagents

A total of 24 6–7-week-old healthy male Wistar rats (SPF grade) with body weights of 160–180 g was purchased from Vital River Laboratory Animal Co. Ltd. (Beijing, China). The rats were housed in a clean room at a temperature of 23 ± 2 °C and a humidity of 50 ± 5% with a 12 h alternating light and dark cycle. They were permitted free access to food and water. All animal experiments were performed according to the National Institutes of Health Guidelines for the Care and Use of Laboratory Animals and were approved by the Animal Care and Use Committee of Shandong Province, China. ETD was provided by national engineering and technology research center of chirality pharmaceutical, Lunan Pharmaceutical Group Co. Ltd.

### Animal treatment

Collagen-induced arthritis (CIA) was induced in rats by intradermal injections in right hindfoot base with 100 μg of bovine collagen type II (CII) (2 mg. mL^− 1^) (Chondrex, Inc., USA) emulsified in complete Freund’s adjuvant (CFA) (2 mg· mL^− 1^; Chondrex, Inc., USA). Eight rats served as control group by intradermal injections in right hindfoot base with saline. Two weeks after immunization, 16 rats with the right heel width>12 mm were selected as the successful model rats, they were randomly divided into two groups (*n* = 8). One group was considered as vehicle- CIA group (V) that received vehicle (0.5% sodium carboxymethyl cellulose, CMC-Na) (n = 8); another group was ETD-CIA group (E) received ETD (5 mg·kg^− 1^) daily as a single dose by gavage for 7 months (n = 8), ETD powder was added into 0.5% sodium carboxymethylcellulose and homogenized for administration. 0.5% CMC-Na was given to the control group (C) (n = 8). All outcome measurements were made by observers blinded to treatment group.

At the end of the experiments, the animals were killed after anesthetization with an intraperitoneal injection of sodium pentobarbital (50 mg· kg^− 1^). Blood was taken from abdominal main vein. The serum was separated from the blood samples by centrifugation at 1000 g for 10 min at 4 °C and taken for biochemical analysis. Synovium of right posterior knee joint in each group were taken after bloodletting, three synovium tissues stored at − 80 °C for further proteomics analysis, and five synovium tissues were fixed in 10% formalin for morphological analysis.

### Assessment of joint arthritis

The articular index was calculated at 0, 0.5, 1, 2, 3, 4, 5, 6 and 7 months after ETD treatment. The followed scoring system were used to assess the severity of arthritis: 0, normal, no evidence of swelling; 1, mild swelling of the toe joint; 2, moderate swelling extending from the toe joint to the ankle; 3, severe swelling extending from the ankle to metatarsal joints; and 4, extreme swelling and deformation of toe joint, the ankle and metatarsal joints. The cumulative score for all four paws of each rat was used as arthritis score (maximum of 16 per rat) to represent overall disease severity and progression. The right heel width was measured with caliper to quantify the swelling in all groups.

### Pain behavior

Punctate allodynia was measured by Ugo Basile Dynamic Plantar Aesthesiometer 37,450. At each paw withdrawal, the Dynamic Plantar Aesthesiometer automatically detects and records actual force at the time of paw withdrawal reflex, and the force is used to indicate the pain threshold.

### HE and Masson staining

Fixed synovium tissue were processed and embedded in tissue embedding rings (Tissue-Tek® TEC™ 5.Sakura Fine tek Japan CO., Ltd). Sections of 4 μm in thickness were subjected to haematoxylin and eosin (HE) staining by automatic staining machine (Leica ST5020). Paraffin sections (4 μm) were carried out for Masson staining. Masson staining kit (Nanjing Jiancheng Bioengineering Institute, Nanjing, Jiangsu, China) was used for Masson staining according to the manufacturers’ instructions. Under the light microscope, the nuclei were dark blue, collagen fibers, cartilage and mucus were blue, muscle fibers, cellulose and red blood cells were red. Slides were viewed and captured by PannoramicViewer1.15.4 (3DHISTECH. Ltd). Synovitis evaluation was refined according to the Krenn’s score [99]. Assessing in four components: inflammatory cell infiltration, synovial cells proliferation, neovascularization and fibrous tissue proliferation. Each item was evaluated by two blind observers using a subscale of 0–4 points, where 0 indicated absence, 1 mild, 2 moderate, 3 severe and 4 extreme. The total synovitis score (TSS) was calculated by adding the individual scores for each component.

### Serum biochemical analysis

The total biochemical indexes of serum were detected using BS-800 automatic biochemistry analyzer (Shenzhen Mindray Bio-Medical Electronics CO., LTD. China).

### Proteomic analysis

The total protein in the sample was extracted, protein concentration was determined by BCA method. 10 μg protein of each sample was separated by 12% SDS-PAGE. The digested peptides were desalted by C18-Reverse-Phase SPE Column. After being desalted, the peptides were separated by a C18 column (50 cm × 75 μm) on an EASY-nLCTM 1200 system (Thermo, USA) and lyophilized for mass spectrometry. All analyses were performed by a Q-Exactive HF mass spectrometer (Thermo, USA) equipped with a Nanospray Flex source (Thermo, USA).

The machine signal is transformed into peptide and protein sequence information by matching the mass spectrum output with the theoretical spectrum generated by fasta library, and then the spectrum library is established by combining the sequence information, peptide retention time and fragment ion information, so as to facilitate the subsequent DIA and PRM analysis. The original LC-MS/MS files are imported into Spectronaut Pulsar software to search and build the database. Briefly, LC-MS/MS identification were consisted of three parts. Firstly, the traditional DDA (Data-dependent Acquisition) method is used to establish the protein spectrum library. Secondly, the mass spectrum data of each sample are collected by DIA technology. At last, the mass spectrum data of candidate proteins are verified by PRM technology.

The original data of DIA is processed by Spectronaut Pulsar software. The proteins of interest to be verified were determined based on the differential protein results of DIA. Import the target proteins list into SpectroDive software (v10.1), match the DDA Library, and the software will automatically calculate the mass charge ratio of the theoretical peptide sequence of the target protein, and retain the unique peptide that meets the conditions. Export the above list and set up a mix pre scan. The pre scanned raw data was imported into SpectroDive software, and the retention time was corrected according to iRT standard peptide. Set the scheduled method and export it. Import the exported list into the inclusion list of xcalibur - PRM method editing module.

### Data and statistical analysis

All experimental data obtained from rats were expressed as mean ± SD. A one-way repeated measure analysis of variance (ANOVA) and a log-rank test were used to determine the significance of the differences in the articular index, TSS of pathological section, biochemical analysis and PRM results, respectively.

For DIA data, difference screening conditions: foldchange≥1.15 or ≤ 0.85 times and *p*-value<0.05. R package was used for the bioinformatics analysis of differentially expressed proteins, and these analyses included KOG function annotation, Go enrichment analysis, KEGG pathway classification and protein interaction analysis. Go enrichment analysis was also carried out to enrich high-level functions in three categories. Protein-protein interaction (PPI) analysis was performed using Metascape online analysis (https://metascape.org/) and String online database (https://string-db.org/).

For PRM, quantitative results of target peptide, the quantitative information of the target peptide was derived. The quantitative value of the protein was calculated by the mean peptide quantity built in the software, and was used for statistical analysis between groups.

## Supplementary Information

**Additional file 1: Supplement Fig 1.** The heap maps of differentially expressed proteins.

**Additional file 2: Supplement Table 1.** The DDA library of the total identified proteins.

**Additional file 3: Supplement Table 2.** The list of total identified proteins.

## Data Availability

The data is available with the corresponding author and will be provided upon the legitimate request.

## Abbreviations

AP: Alternative pathway
ApoE: Apolipoprotein E
Bzw2: Basic leucine zipper and W2 domains 2
Carhsp1: Calcium-regulated heat stable protein 1
Cdc37: Cell division cycle 37
CIA: Collagen induced arthritis
Col11a1: Collagen alpha-1(XI) chain
Col4a1: Collagen type IV alpha 1 chain
Colecl2: Collectin-12
COX: Cyclooxygenase
CP: Classical pathway
Creb1: Cyclic AMP-responsive element-binding protein 1
Ctsd: Cathepsin D
Ctsh: Cathepsin H
DEP: Differential proteins
DIA: Data independent acquisition mass spectrometry
Eif4b: Eukaryotic initiation factor 4B
Elavl1: Embryonic Lethal Abnormal Vision-Like Protein 1
ETD: Etodolac
Fg: Fibrinogen
Gpx1: Glutathione peroxidase
Hnrnph1: Heterogeneous nuclear ribonucleoprotein H
HnrnpL: Heterogeneous nuclear ribonucleoprotein L
Hspa12b: Heat shock protein family A (Hsp70) member 12B
Hspa1a: Heat shock 70 kDa protein 1A
Hspa2: Heat shock-related 70 kDa protein 2
Hspb7: Hsp27
Klkb1: Plasma kallikrein
Lamp1: Lysosome-associated membrane glycoprotein 1
Lum: Lumincan
Mcts1: Multiple copies in T-cell malignancy 1
MS: Mass spectrometry
Nfic: Nuclear factor I-C
NSAIDs: Non-steroidal anti-inflammatory drugs
PG: Prostaglandin
PK: Plasma prokallikrein
PRM: Parallel reaction monitoring
Ptbp1: Polypyrimidine tract-binding proteins 1
Ptges3: Prostaglandin E synthase 3
Pura: Transcriptional activator protein Pur-α
RA: Rheumatoid arthritis
RBP: RNA binding protein
RF: Rheumatoid factor
Ruvbl1: RuvB-like 1
Septin9: Cytoskeleton interacting protein septin9
U1–70K: U1 small nuclear ribonucleoprotein 70 kDa
Snrpd3: Small nuclear ribonucleoprotein Sm D3
Srrm2: Serine/arginine repetitive matrix protein 2
Srsf7: Serine/arginine-rich splicing factor 7
Tgfbi: Transforming growth factor-β-inducible protein
Tmsb4x: Thymosin β4
Top2a: Topoisomerase IIα
U2af2: U2 snRNP auxiliary factor large subunit
Wars: Tryptophanyl-tRNA synthetase
Yars: Tyrosyl- tRNA synthetase
