# Supplementary figures and images for: Etodolac improves collagen induced rheumatoid arthritis in rats by inhibiting synovial inflammation, fibrosis and hyperplasia
Source: Mol Biomed. 2021 Oct 25;2:33. doi: 10.1186/s43556-021-00052-1 (PMC8607370; doi:10.1186/s43556-021-00052-1)

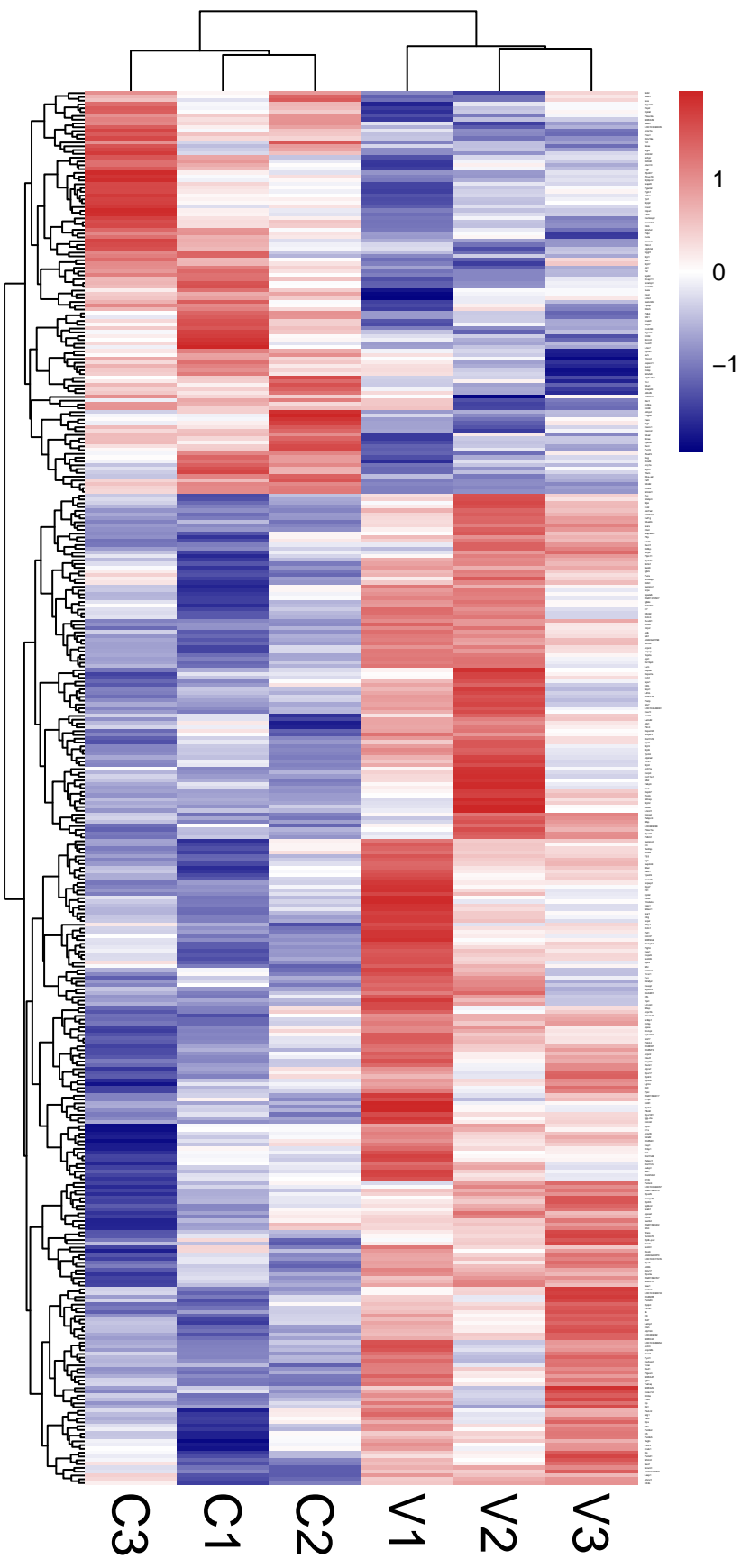

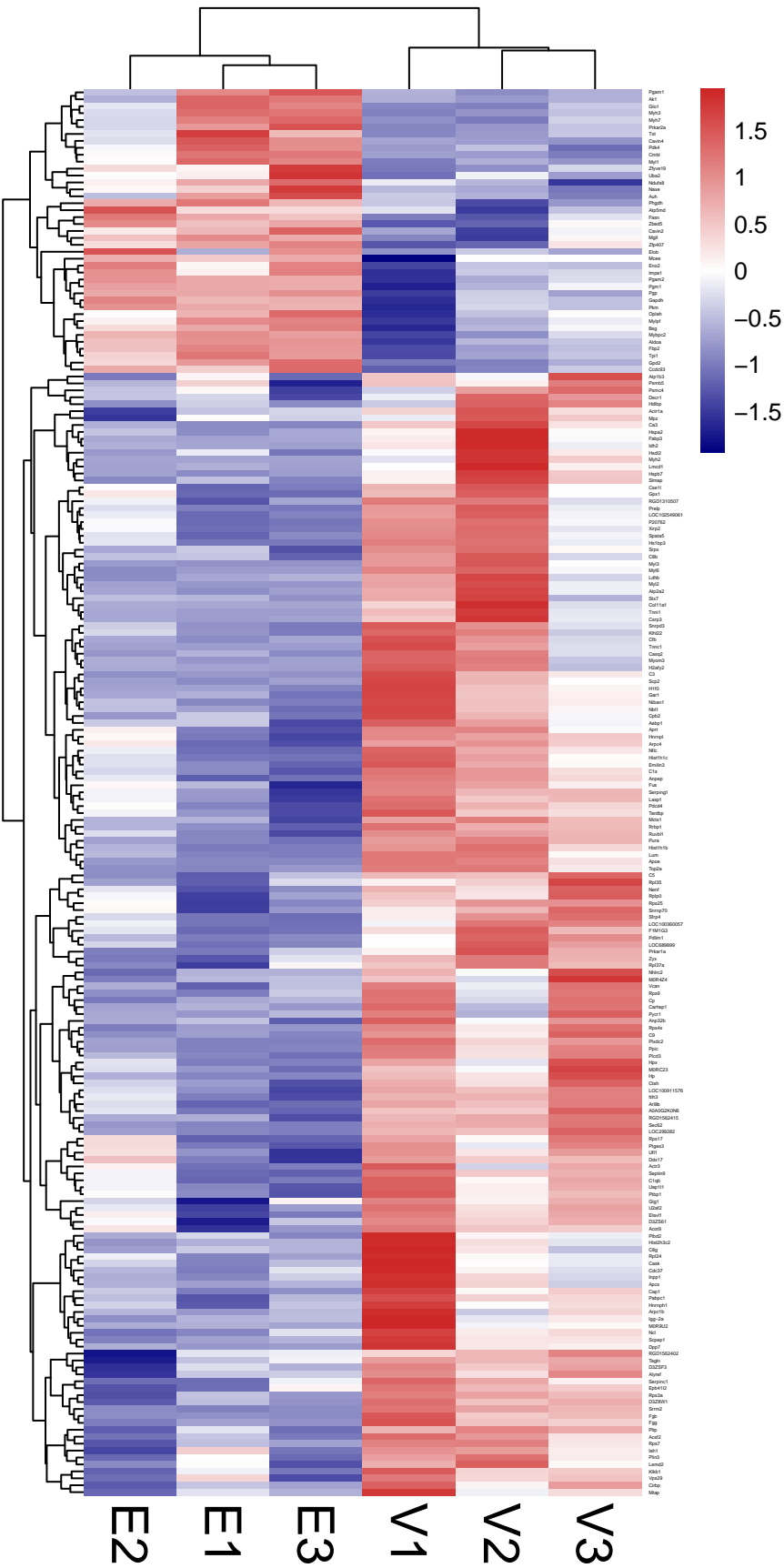

Supplement: Supplementary file 1 — Additional file 1: Supplement Fig 1. The heap maps of differentially expressed proteins. [file 43556_2021_52_MOESM1_ESM.pdf]
